# Compulsory Registration of Nurses and the Untrained: A Suggestion

**Published:** 1920-01-03

**Authors:** 


### COMPULSORY REGISTRATION OF NURSES AND THE UNTRAINED: A SUGGESTION.

*To the Editor of* THE HOSPITAL.

SIR,—The objection to the compulsory registration of nurses lies in the fact that a great deal of attendance on the sick can be carried out by untrained persons, and that their employment is both legitimate and useful; yet the control of the untrained is equally necessary with that of the trained. Here is a suggestion. Let every untrained sick attendant be *licensed as such* (the term "nurse" being confined to trained persons on the State Register. The licences to be granted and the roll kept by the medical officer of health for the district in which the sick attendant desires to practise. The applicant to produce proof of age (twenty-one and over) and good character, accompanied by a certificate from a registered medical practitioner stating that he or she is a fit and competent person to undertake the duties of an untrained attendant on the sick. Licences, like the certificates of registration, to be renewed annually. Registration of trained nurses and licensing of sick attendants to be compulsory; nursing and attendance on the sick for gain to be prohibited to the unregistered and unlicensed, and punishable by fine, or, if repeated, by imprisonment. Licences to be suspended for proved misconduct or incompetence.

A PROVINCIAL MATRON.

